# Dual-Mode Scandium-Aluminum Nitride Lamb-Wave Resonators Using Reconfigurable Periodic Poling

**DOI:** 10.3390/mi13071003

**Published:** 2022-06-26

**Authors:** Sushant Rassay, Dicheng Mo, Roozbeh Tabrizian

**Affiliations:** Electrical and Computer Engineering Department, University of Florida, Gainesville, FL 32603, USA; sushantrassay@ufl.edu (S.R.); dicheng.mo@ufl.edu (D.M.)

**Keywords:** ferroelectric, scandium–aluminum nitride, Lamb-wave resonators, complementary switchable

## Abstract

This paper presents the use of ferroelectric behavior in scandium–aluminum nitride (Sc_x_Al_1−x_N) to create dual-mode Lamb-wave resonators for the realization of intrinsically configurable radio-frequency front-end systems. An integrated array of intrinsically switchable dual-mode Lamb-wave resonators with frequencies covering the 0.45–3 GHz spectrum. The resonators are created in ferroelectric scandium–aluminum nitride (Sc_0.28_Al_0.72_N) film and rely on period poling for intrinsic configuration between Lamb modes with highly different wavelengths and frequencies. A comprehensive analytical model is presented, formulating intrinsically switchable dual-mode operation and providing closed-form derivation of electromechanical coupling ($k_{t}^{2}$) in the two resonance modes as a function of electrode dimensions and scandium content. Fabricated resonator prototypes show $k_{t}^{2}$s as high as 4.95%, when operating in the first modes over 0.45–1.6 GHz, 2.23% when operating in the second mode of operation over 0.8–3 GHz, and series quality factors ($Q_{s}$) over 300–800. Benefiting from lithographical frequency tailorability and intrinsic switchability that alleviate the need for external multiplexers, and large $k_{t}^{2}$ and Q, dual-mode Sc_0.28_Al_0.72_N Lamb-wave resonators are promising candidates to realize single-chip multi-band reconfigurable spectral processors for radio-frequency front-ends of modern wireless systems.

## 1. Introduction

Scandium–aluminum nitride (Sc_x_Al_1−x_N) has recently emerged as a transforming piezoelectric material for creation of high-performance electroacoustic resonators and filters. Benefiting from the large piezoelectric coefficients that only increase with scandium content [1], Sc_x_Al_1−x_N enables the radical enhancement of electromechanical coupling ($k_{t}^{2}$) in electroacoustic resonators. This facilitates the creation of radio-frequency (RF) filters with significantly lower loss and higher bandwidth compared to AlN counterparts [2,3,4,5]. Besides the $k_{t}^{2}$ enhancement, the recent discovery of ferroelectricity in Sc_x_Al_1−x_N [6] has initiated extensive research efforts for the creation of configurable RF components, such as varactors, and tunable and switchable resonators and filters [7,8,9,10,11,12]. These components are of particular interest for emerging wireless communication systems that require multi-band adaptive operation over a wide frequency spectrum [13,14].

Currently, the RF front-end of wireless systems rely on a large set of AlN thickness-extensional bulk acoustic wave (BAW) filters that are arrayed at the board level using external multiplexers, to enable spectral processing over the 0.4 GHz to 6 GHz spectrum [13,15,16]. The frequency of BAW filters is defined by the thickness of the metal–piezoelectric–metal stack and cannot be tailored with lithography. This imposes the need for a large number of separately packaged filter chips to address the numerous bands for different wireless applications and protocols.

Lamb-wave AlN resonators have been extensively explored as an alternative to BAW, as they provide lithographical frequency scalability and enable the integration of multi-band filters on a single chip [17]. However, the lower $k_{t}^{2}$ of Lamb-wave resonators compared to their BAW counterparts, and the resulting limitation in maximum attainable filter bandwidth has set a barrier for their adoption in RFFE. The lower $k_{t}^{2}$ of Lamb-wave resonators is due to the smaller transverse piezoelectric coefficient ($e_{31}$) compared to longitudinal ($e_{33}$) in AlN films.

However, the $k_{t}^{2}$ shortcoming in Lamb-wave resonators can be resolved considering the substantial increase in $e_{31}$ with sufficiently high Sc doping that enables the realization of Sc_x_Al_1−x_N Lamb-wave resonators with $k_{t}^{2}$ on par with or exceeding AlN BAW resonators [2,3,5]. Further, the ferroelectricity in Sc_x_Al_1−x_N provides new opportunities for the intrinsic and on-chip reconfiguration of Lamb-wave resonators, to further reduce the number of filters and external switches, and their corresponding load on RFFE footprint, power consumption, and latency [18]. Another application of ferroelectricity is the use of polarization engineering to tailor excitable resonance modes for the performance optimization of electroacoustic resonators and filters. Polarization engineering has been previously demonstrated in lithium niobate electroacoustic devices for improving the response of an acoustically coupled filter [19], in the extreme frequency scaling of a resonator by enabling excitation of higher harmonics [20], and in the creation of acoustic stop-bands in waveguides [21]. In these efforts, polarization tailoring is applied as a part of the fabrication process. This approach does not allow the use of on-chip polarization tuning for the dynamic reconfiguration of device operation that is highly desirable for adaptive spectral processing applications.

In this work, we demonstrate high-$k_{t}^{2}$ dual-mode intrinsically switchable Sc_0.28_Al_0.72_N Lamb-wave resonators. Intrinsic switchability and dual-mode operation are realized by the periodic poling of Sc_0.28_Al_0.72_N, using pulsed switching, to enable the selective excitation of Lamb modes with different wavelengths and frequencies. Dual-mode Lamb-wave resonators with frequencies covering the entire ultra-high-frequency regime are implemented in the same batch, and their intrinsic switchability and dual-mode operation are analytically formulated and experimentally verified.

## 2. Concept

**Figure 1:**
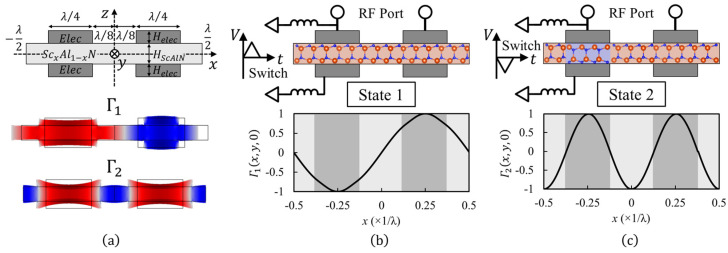
(**a**) Cross−sectional schematic of unit-cell in the Lamb-wave resonators, with strain mode−shapes when operating in $\Gamma_{1}$ and $\Gamma_{2}$ modes. (**b**) Operation State 1 (unified polarization): polarization under all the IDTs is in same direction, enabling the high-$k_{t}^{2}$ excitation of $\Gamma_{1}$ while $\Gamma_{2}$ mode is turned off. (**c**) Operation State 2 (alternating polarization): polarization under consecutive IDTs is in the opposite direction, enabling the high-$k_{t}^{2}$ excitation of $\Gamma_{2}$ while $\Gamma_{1}$ mode is turned off.

Lamb-wave resonators are created from cascading unit-cells with patterned interdigitated transducers (IDT) (Figure 1a).

Considering the resonator as a waveguide extended in the *x*-axis direction, the mechanical resonance modes correspond to the eigenmodes of the unit cell, when a periodic boundary condition is applied:

(1) $$\Gamma_{i}\left(x,y,z\right)=\Gamma_{i}\left(x+\lambda ,y,z\right)$$

Here, $\Gamma_{i}\left(x,y,z\right)$ is the strain mode–shape function, and λ is the unit-cell length in x-axis direction. Figure 1a shows the COMSOL-simulated deformation mode–shape for two different eigenmodes, corresponding to the zeroth-order symmetric Lamb waves (i.e., $S_{0}$) propagating in the x-axis direction, that optimally match the lateral electric-field excitation scheme using top-surface IDTs. The simulation is performed for a unit-cell with Sc_x_Al_1−x_N thickness of 200 nm, molybdenum (Mo) bottom electrode and IDTs with a thickness of 100 nm, and λ of 6 mm. These modes benefit from the efficient electromechanical excitation enabled by the large $e_{31}$ in Sc_x_Al_1−x_N. This can be formulated using the excited electric displacement in the two modes ($D_{S_{0},i},\;i=1,2$) as:

(2) $$D_{S_{0},i}\left(x\right)=e_{31,eff}\Gamma_{S_{0},i}$$

Here, $e_{31,eff}$ is the effective transverse piezoelectric coefficient that is linearly proportional to the normalized instantaneous polarization $P_{inst}\left(x\right)$, due to the ferroelectric characteristic in Sc_0.28_Al_0.72_N and is formulated as:

(3) $$e_{31,eff}=e_{31}P_{inst}\left(x\right)$$

$P_{inst}\left(x\right)$ can be spatially tuned between 1 (i.e., nitrogen polar) or −1 (i.e., metal polar) by the application of proper polarization-switching pulses to Sc_0.28_Al_0.72_N, between each IDT finger and the bottom electrode. The motional charge per unit length of the y-axis ($Q_{m,i}$) excited between the two IDT fingers in the unit-cell is derived from:

(4) Qm,i=12∑j=1,2((−1)j∫xelec,jxelec,j+λ4DS0,i(x)dx)

Assuming a similar acoustic velocity in metal electrodes and Sc_0.28_Al_0.72_N, and infinite dimension of the waveguide in y-axis direction, the $S_{0}$ mode–shapes are:

(5) $$\begin{array}{c} \Gamma_{1}\left(x,y,z\right)≅\sin\left(\frac{2\pi x}{\lambda }\right),\\ \Gamma_{2}\left(x,y,z\right)≅\cos\left(\frac{4\pi x}{\lambda }\right) \end{array}$$

The coupling coefficient ($k_{t,i}^{2}$) of the ith $S_{0}$ mode (i=1,2) is formulated from mechanical and electrical energies as [22]:

(6) $$k_{t,i}^{2}≅\frac{\frac{1}{2}\frac{Q_{m,i}^{2}}{C_{0}}}{\frac{1}{2}\frac{Q_{m,i}^{2}}{C_{0}}+\int_{0}^{H_{ScAlN}}\int_{0}^{\lambda }\frac{c_{11}}{2}{\left(\Gamma_{S_{0},i}\right)}^{2}dxdz+4\int_{0}^{H_{elec}}\int_{\frac{\lambda }{4}-\frac{W_{f}}{2}}^{\frac{\lambda }{4}+\frac{W_{f}}{2}}\frac{E_{elec}}{2}{\left(\Gamma_{S_{0},i}\right)}^{2}dxdz}$$

Here, $H_{ScAlN}$ and $H_{elec}$ are the thickness of the piezoelectric film and electrodes, respectively. $c_{11}$ and $E_{elec}$ are, respectively, the elastic constants of the piezoelectric film and electrodes in the wave-propagation direction. $W_{f}$ is the IDT finger width. $C_{0}$ is the capacitance between the two IDT fingers, per unit length in the y-axis direction, and is approximated as:

(7) $$C_{0}=\frac{\epsilon_{33}W_{f}}{2H}$$

Here, $\epsilon_{33}$ is the piezoelectric film permittivity. Replacing Equations (4) and (5) in Equation (6), $k_{t,i=1,2}^{2}$ is simplified to:

(8) $$k_{t,i}^{2}={\left(\frac{P_{inst,1}-{\left(-1\right)}^{i}P_{inst,2}}{2}\right)}^{2}\frac{\frac{4}{\pi^{2}}K_{31}^{2}\alpha_{i}}{\beta_{i}+\frac{4}{\pi^{2}}K_{31}^{2}\alpha_{i}}$$

Here, $\alpha_{i}$ is a scaling factor corresponding to the relative width of IDT fingers to unit-cell length, calculated as:

(9) $$\alpha_{i}=\frac{\lambda }{i^{2}W_{f}}{\left(\sin\left(\frac{i\pi W_{f}}{\lambda }\right)\right)}^{2}\;$$

$\beta_{i}$ is a scaling factor representing the relative energy distribution in overall unit cell and the piezoelectric layer, calculated as:

(10) $$\beta_{i}=\frac{c_{11}H_{ScAlN}+\frac{2\alpha_{i}W_{f}}{\lambda }E_{elec}H_{elec}}{c_{11}H_{ScAlN}}$$

$P_{inst,j}\;\left(j=1,2\right)$ are the net polarization of Sc_0.28_Al_0.72_N under the two IDT fingers in the unit-cell, and $K_{31}^{2}$ is the transverse piezoelectric coupling constant formulated as:

(11) $$K_{31}^{2}=\frac{e_{31}^{2}}{\epsilon_{33}c_{11}}$$

Considering Equation (8), two complementary polarization states exist where either the first (i.e., $\Gamma_{1}$) or the second (i.e., $\Gamma_{2}$) $S_{0}$ mode has the maximum $k_{t}^{2}$. In State 1, wherein the polarizations under both IDT fingers are unified (i.e., all in the same direction: $P_{inst,j}\;\left(j=1,2\right)=\pm 1$), $\Gamma_{1}$ is excited with the maximum $k_{t}^{2}$, while $\Gamma_{2}$ is switched off (i.e., $k_{t,2}^{2}=0$). In State 2, wherein the polarizations under IDT fingers are periodically alternating (i.e., in opposite directions: $P_{inst,1}=-P_{inst,2}=\pm 1$), $\Gamma_{2}$ is excited with the maximum $k_{t}^{2}$, while $\Gamma_{1}$ is switched off (i.e., $k_{t,1}^{2}=0$). Figure 1b,c shows the complementary operation states corresponding to different polarization configurations and the x-axis strain mode–shape function for the active mode.

**Figure 2:**
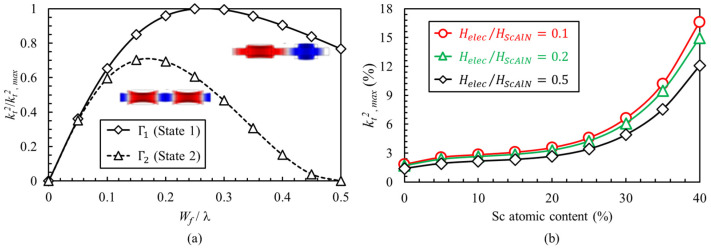
(**a**) Normalized $k_{t}^{2}$ of $\Gamma_{1}$ and $\Gamma_{2}$ modes across different electrode finger widths and for arbitrary Sc content and electrode thickness. (**b**) Maximum achievable $k_{t}^{2}$ of $\Gamma_{1}$ mode for different Sc contents and electrode thicknesses.

The complementary operation enables intrinsic switching of the resonator between fundamental and second harmonics of Lamb modes, with a frequency ratio near 2. Considering Equations (8)–(10), the relative magnitude of $k_{t}^{2}$ for these modes depends on the electrode finger width. Figure 2a shows the normalized $k_{t}^{2}$ of $\Gamma_{1}$ and $\Gamma_{2}$ modes, across different finger widths, extracted using the presented analytical model. It is evident that the $k_{t}^{2}$ of $\Gamma_{1}$ mode is always higher than that of $\Gamma_{2}$. When using the dual-mode resonator to implement a dual-band bandpass filter, the lower $k_{t}^{2}$ of mode $\Gamma_{2}$ translates into a lower fractional bandwidth. However, the absolute bandwidth of the filter remain nearly the same in either operation modes, considering the higher frequency of $\Gamma_{2}$ mode. As modern wireless networking protocol applies similar channel bandwidth at different center frequencies (e.g., 40 MHz in both 2.4 GHz and 5 GHz in IEEE 802.11 n), a halved $k_{t}^{2}$ of $\Gamma_{2}$ at a frequency that is nearly twice $\Gamma_{1}$ enables the realization of a dual-band filter with the same absolute bandwidth in both operation states. Figure 2b shows the maximum $k_{t}^{2}$ achievable in mode $\Gamma_{1}$, for resonators created from Sc_x_Al_1−x_N films with Sc content over 0% to 40%. This plot is derived using Equation (8) and for different thicknesses of the metal electrode relative to Sc_x_Al_1−x_N film. It is evident that, for Sc_x_Al_1−x_N films exceeding 30% Sc content, assuming 0.1 relative thickness of electrodes, the $k_{t}^{2}$ of both modes exceeds the 6% typical value in AlN BAW resonators. It should also be noted that, considering the very large polarization-switching fields in Sc_x_Al_1−x_N, thinner films are desirable to enable a configuration between the two modes with reasonable voltages. Therefore, opting for 0.1 relative thickness of metal films may result in excessive electrode loss. In this work, the resonators are implemented in ~200 nm Sc_0.28_Al_0.72_N films, and ~100 nm Mo electrodes (i.e., 0.5 relative electrode thickness) are used.

## 3. Fabrication Process

**Figure 3:**
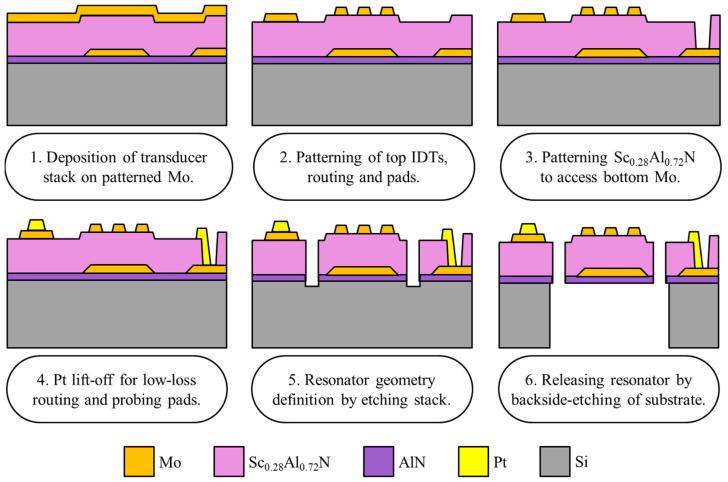
Fabrication process flow of Sc_0.28_Al_0.72_N Lamb-wave resonators.

Figure 3 shows the fabrication process for the creation of the dual-mode Sc_0.28_Al_0.72_N Lamb-wave resonators. The process consists of the DC sputtering of a 100 nm Mo layer atop a 30 nm AlN film that serves as a seed for (110)-textured growth of Mo film [23]. The Mo layer is then patterned using a boron trichloride (BCl_3_) gas-based recipe in a reactive-ion-etching inductively coupled plasma (RIE-ICP) system. Prior to etching, a tapered photoresist mask was developed via the proximity exposure method. The resulting tapered photoresist profile thus enabled the formation of tapered sidewalls in the Mo layer, which promoted the crack-free growth of the subsequent Sc_0.28_Al_0.72_N layer. Following this, a highly crystalline *c*-axis-oriented 200 nm Sc_0.28_Al_0.72_N layer was deposited using reactive magnetron sputtering from segmented scandium–aluminum targets [24]. Finally, atop this, a ~120 nm thick Mo layer was deposited, to serve as the top electrode for the resonator.

**Figure 4:**
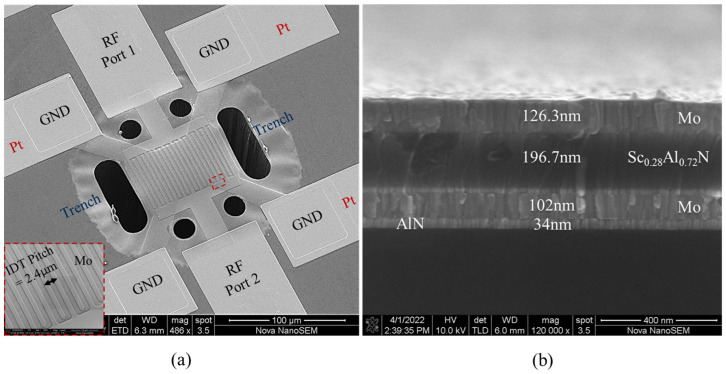
(**a**) SEM image of Sc_0.28_Al_0.72_N Lamb−wave resonator. The inset shows the IDT with a 2.4 µm pitch size. (**b**) Cross−sectional SEM image of the resonator, detailing constituent-layer thicknesses.

After the deposition of the transducer stack, the top Mo layer is patterned using SF_6_ in the RIE-ICP system to form IDTs. Next, to access the bottom electrode, Sc_0.28_Al_0.72_N is patterned using timed Cl_2_ dry-etch in a RIE-ICP system. This is followed by the deposition of 500 nm thick Cr/Pt through lift-off, to create low-loss lines and probing pads. After metallization, the lateral geometry of the resonator is formed by etching trenches using a high-power Cl_2_/BCl_3_-based recipe in the RIE-ICP system, wherein low-frequency PECVD SiO_2_ is used as a hard-mask for the etching process. Finally, the device is released from the backside of the silicon substrate by deep reactive ion etching (DRIE). Figure 4a shows the SEM image of the Lamb-wave resonator and highlights the patterned IDTs. Figure 4b shows the cross-sectional SEM image of the resonator stack, highlighting the thickness of the constituent layers.

## 4. Characterization

Dual-mode Sc_0.28_Al_0.72_N Lamb-wave resonators with different IDT pitch sizes were measured to identify their admittance and switching behavior. Ferroelectric polarization hysteresis loop measurement and the periodic poling of the Sc_0.28_Al_0.72_N were performed using a Radiant PiezoMEMS ferroelectric tester. The resonators’ RF performance was measured using a Keysight N5222A PNA vector network analyzer, along with a short-open-load-through (SOLT) calibration procedure enabled by a CS-5 calibration kit from GGB Industries INC.

### 4.1. Ferroelectric Characterization

**Figure 5:**
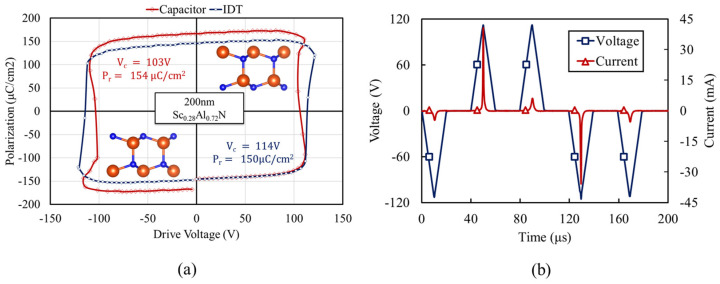
(**a**) Polarization−voltage (P−V) hysteresis loop measured at an IDT port and a 100 μm×100 μm capacitor. (**b**) The measured instantaneous current at the IDT port, upon the application of a 45 kHz triangular PUND pulse sequence, highlighting polarization reversal in Sc_0.28_Al_0.78_N film.

To identify the switching voltage, polarization hysteresis loops were measured using 100 kHz bipolar triangular pulses with 125 V amplitude. Figure 5a shows the hysteresis loop measured at an IDT port and is compared with the loop measured for a 100 μm×100 μm capacitor. The slight degradation of the loop measured at the IDT port, defined by a lower remanent polarization and higher coercive field, may correspond to the nonuniform distribution of the electric field at excessive edges of IDTs. A coercive voltage of 114 V is extracted for the IDT port, identifying the required voltage for the periodic poling of Sc_0.28_Al_0.72_N to switch resonator operation between $\Gamma_{1}$ and $\Gamma_{2}$ modes. Figure 5b shows the instantaneous current measured at the IDT port, upon the application of a 45 kHz negative positive-up-negative-down (PUND) pulse sequence with a 112 V amplitude. The large instantaneous current induced upon a change in the sign of deriving voltage pulse indicates the polarization inversion of Sc_0.28_Al_0.72_N between metal- and nitrogen-polar states. A similar waveform, with slightly lower voltage of 110 V, is used for periodic poling and the intrinsic switching of the resonator between the two operation states. Opting for lower voltage enables the observation of resonator admittance evolution during the transition between the two operation states and the corresponding complementary excitation and suppression of $\Gamma_{1}$ and $\Gamma_{2}$ modes.

### 4.2. RF Characterization

**Figure 6:**
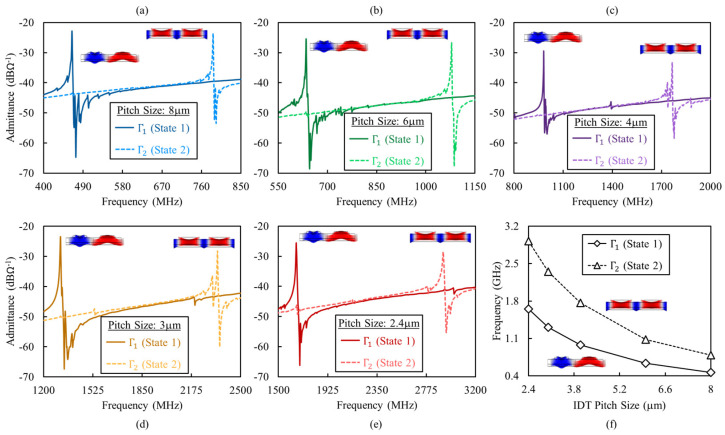
Measured admittance of dual−mode Sc_0.28_Al_0.72_N Lamb−wave resonators when operating in either of the complementary switchable states defined by periodic poling procedure. The admittances are shown for resonators with (**a**) 8 mm, (**b**) 6 mm, (**c**) 4 mm, (**d**) 3 mm, and (**e**) 2.4 mm IDT pitch sizes. (**f**) The frequency of $\Gamma_{1}$ and $\Gamma_{2}$ modes for different IDT pitch sizes, highlighting the coverage of the 0.45−3 GHz spectrum.

The admittance of resonators was extracted from the measured reflection coefficient (i.e., *S*_11_). One-port measurements were performed through the application of a signal between the two IDT ports while keeping the bottom electrode floating. The intrinsic switching of the resonators between the two operation states is performed by applying pulsed poling voltages between one of the IDT ports and the bottom electrode. Figure 6a–e shows the measured admittances for resonators with different IDT pitch sizes ranging over 2.4 mm to 8 mm. For each resonator, the measured admittances are shown when operating in each state. The complementary switchable dual-mode operation in $\Gamma_{1}$ and $\Gamma_{2}$ modes is evident. This is enabled by the pulsed periodic poling of Sc_0.28_Al_0.72_N under one of the IDT ports. Figure 6f shows the frequency of the $\Gamma_{1}$ and $\Gamma_{2}$ modes for different IDT pitch sizes, highlighting the coverage of the 0.45–3 GHz spectrum.

**Figure 7:**
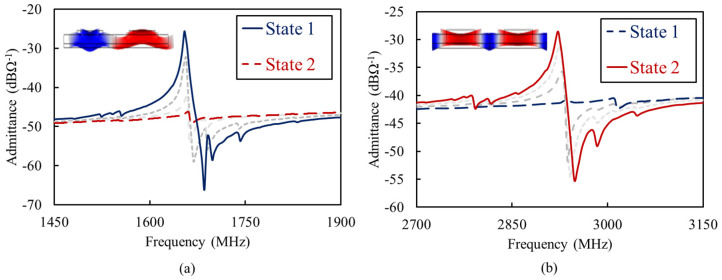
Short−span measured admittance of the Lamb−wave resonator with an IDT pitch size of 2.4 mm when operating in (**a**) State 1 and (**b**) State 2. The intermediate admittance plots, shown in dashed gray line, highlight the transition between the two operation states.

Figure 7 shows the short-span admittance of the Sc_0.28_Al_0.72_N resonator with 2.4 mm IDT pitch size, around $\Gamma_{1}$ and $\Gamma_{2}$ resonance frequencies. The evolution of admittance upon the application of three −110 V 45 kHz monopolar poling pulses, between one of IDT terminals and the floating bottom electrode, is evident. Starting from the pristine film with uniform polarization, the application of the first and second poling pulses to one of the IDT terminals results in periodic, yet partial, polarization switching. This translates to the gradual suppression of mode $\Gamma_{1}$ and the emergence of mode $\Gamma_{2}$. After the third pulse, when the polarization of all the domains under the corresponding IDT terminal are fully reversed, $\Gamma_{1}$ is fully suppressed, while $\Gamma_{2}$ has emerged with its maximum $k_{t}^{2}$. The reversibility of this procedure is verified by the application of three 45 kHz monopolar poling pulses, but with a 110 V amplitude.

**Figure 8:**
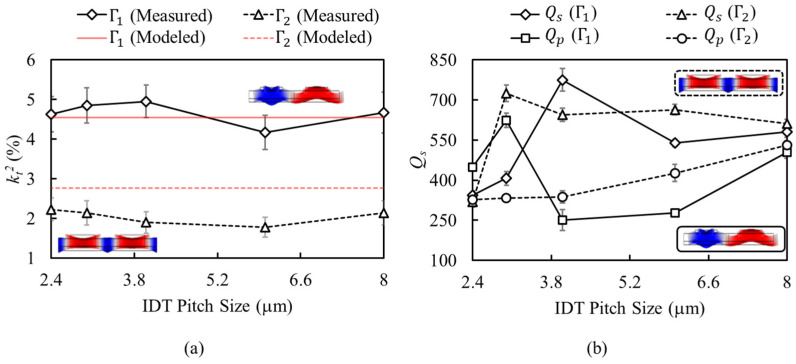
(**a**) Measured $k_{t}^{2}$ of the two modes for resonators with different IDT pitch sizes, in comparison with values extracted from analytical model. (**b**) Measured *Q_s_* and *Q_p_* of the two modes, for resonators with different IDT pitch sizes. For each IDT pitch size, the $k_{t}^{2}$, *Q_s_*, and *Q_p_* are the average of values measured from ten resonators across the 4-inch substrate.

Figure 8 shows the measured $k_{t}^{2}$, $Q_{s}$, and $Q_{p}$ (i.e., Q at series and parallel resonance) of resonators with different IDT pitch sizes over 2.4 mm to 8 mm, covering 0.45 GHz to 3 GHz in two operation states. For each IDT pitch size, ten resonators are measured across the wafer. The resonators $k_{t}^{2}$ and Q are extracted from the admittances using [25,26]:

(12) $$k_{t}^{2}=\frac{\pi^{2}}{8}\left(\frac{f_{p}{}^{2}-f_{s}{}^{2}}{f_{s}{}^{2}}\right),\;Q=\frac{f}{2}\left|\frac{\partial \varphi_{Y}}{\partial f}\right|$$

Here, $f_{s}$ and $f_{p}$ are the frequencies of series and parallel resonance modes, and $\varphi_{Y}$ is the admittance phase. The measured $k_{t}^{2}$ is compared with the values extracted from the presented analytical model.

$k_{t}^{2}$ mean values over 4.17% to 4.95% are measured for the resonators when operating in $\Gamma_{1}$ mode (i.e., State 1 configuration). This is very close to the 4.58% value extracted from the analytical model. When operating in $\Gamma_{2}$ mode (i.e., State 2 configuration), $k_{t}^{2}$ mean values over 1.78% to 2.23% are measured for the resonators. The measured $k_{t}^{2}$ are slightly lower than the 2.77% extracted from the analytical model. This discrepancy may be attributed to the nonuniformity of strain field across the transducer thickness, which is aggravated at higher frequency (i.e., smaller wavelength) and results in higher energy concentration in Mo electrodes. Spurious modes are generated due to the two-dimensional nature of Lamb-wave propagation in a transducer membrane. Ideally, having a structure with infinitely long IDTs enables the creation of a spurious-free S_0_ resonator. In practice, the finite length of the IDTs and the mechanical boundary at the substrate-anchoring region result in the energy localization of Lamb waves with a non-zero wavenumber in the IDT length direction. These waves create spurious modes with distribution and frequency defined by IDT length and the mechanical termination of the membrane. The spurious modes can be suppressed through the proper apodization of IDTs to avoid their excitation or by reducing their coupling through charge cancelation [27].

Finally, as Figure 8b suggests, large Q variations, with no clear trend, is observed in resonators with different IDT pitch size. This may correspond to the varying length of IDTs in different resonator designs, and its influence on the energy localization and $Q_{s}$. These variations are also observed in $Q_{p},$ which may be attributed to the non-homogeneous distribution of spurious modes for different IDT dimensions. Opting for optimized IDT length and exploiting apodization techniques enable achieving consistent $Q_{s}$ that are only limited by fundamental material-related energy-dissipation mechanisms [27,28,29]. Considering the $k_{t}^{2}$ and $Q_{s}$ values presented in Figure 8, the maximum figure of merits (i.e., $k_{t}^{2}\times Q_{s}$) of ~38 and ~16 are measured for $\Gamma_{1}$ and $\Gamma_{2}$ modes, when operating in State 1 and State 2, respectively. These values can be further improved by opting for higher Sc content in the Sc_x_Al_1−x_N film or by reducing *W_f_* to ~λ5 to achieve an optimized $k_{t}^{2}$ for both modes.

## 5. Conclusions

This paper presented a new reconfigurable Sc_x_Al_1−x_N Lamb-wave resonator technology based on the use of ferroelectric behavior. Periodic polarization tuning, through interdigitated transducers (IDT), was used for complementary switching between two Lamb modes with highly different wavelengths and frequencies. A comprehensive analytical model was presented to verify the complementary switchable dual-mode operation of the resonator and to provide closed-form formulation to identify the electromechanical coupling ($k_{t}^{2}$) of the two modes as a function of scandium content, IDT electrode thickness, and finger width. The fabrication process for the implementation of a dual-mode Lamb-wave resonator in Sc_0.28_Al_0.72_N film was presented. Prototypes with IDT pitch sizes over 2.4 mm to 8 mm were characterized to identify their switching behavior and RF admittance. Period poling was performed through the application of 110 V 45 kHz triangular pulses between one of the IDT ports and the bottom electrode, enabling successful complementary switching between two modes of operation. $k_{t}^{2}$s over 4.17–4.95%, when operating in the first modes over 0.45–1.6 GHz, and 1.78–2.23% when operating in the second mode of operation over 0.8–3 GHz, were measured. Series quality factors ($Q_{s}$) over 300–800 were extracted for resonators operating in first and second modes over 0.45–3 GHz. The presented dual-mode complementary-switchable Sc_x_Al_1−x_N Lamb-wave resonator technology provides lithographical frequency scaling over the entire ultra-high-frequency GHz bands; large $k_{t}^{2}$ exceeding AlN BAW, once doped with sufficiently high scandium content; and intrinsic switchability to relieve the need for external multiplexers. These highlight a high potential to create single-chip multi-band spectral processors for modern wireless systems.

## Data Availability

Data available on request due to restrictions e.g., privacy or ethical.
